# Differential Effect of Growth on Development between AGA and SGA Preterm Infants

**DOI:** 10.3390/ijerph17093022

**Published:** 2020-04-27

**Authors:** In Gyu Song, Ee-Kyung Kim, Hannah Cho, Seung Han Shin, Jin A. Sohn, Han-Suk Kim

**Affiliations:** 1 National Hospice Center, National Cancer Center, Goyang-si 10408, Korea; pedigms@gmail.com; 2 Department of Pediatrics, Seoul National University Children’s Hospital, Seoul National University College of Medicine, Seoul 03080, Korea; revival421@snu.ac.kr (S.H.S.); kimhans@snu.ac.kr (H.-S.K.); 3 Department of Pediatrics, Korea University Anam Hospital, Seoul 02857, Korea; lovevnhannah@naver.com; 4 Department of Pediatrics, Seoul Metropolitan Government Seoul National University Boramae Medical Center, Seoul National University College of Medicine, Seoul 07061, Korea; jinasohn@hanmail.net

**Keywords:** head circumference, growth, neurodevelopment, preterm infant, small for gestational age

## Abstract

Predicting developmental outcomes with growth measurement would be beneficial for primary healthcare or in developing countries with low medical resources. This study aimed to identify physical growth measures that indicate neurodevelopment in very preterm infants. Preterm infants, born at <32 weeks’ gestation or weighing <1500 g, were included. We calculated the changes in z-score of weight, length, and head circumference (HC) at different time points: birth, postmenstrual age (PMA) 35 weeks, and 4 and 18 months corrected age (CA). We examined the relationship between growth and Bayley-III scores using linear regression. Among 122 infants, HC at 4 months CA and HC growth between PMA 35 weeks and 4 months CA showed a positive correlation with Bayley-III scores in appropriate-for-gestational-age infants (AGAs). Weight and length increases between birth and 18 months CA were also associated with AGAs’ development. In small-for-gestational-age infants (SGAs), only birthweight’s z-score was associated with improved neurodevelopmental outcomes. HC at 4 months CA was an important indicator of favorable neurodevelopmental outcomes, and head growth spurt between PMA 35 weeks and 4 months CA contributed to this benefit in preterm AGAs. The period and indices should be monitored differently for SGAs and AGAs.

## 1. Introduction

Globally, approximately 15 million babies are born preterm (before 37 completed weeks of gestation) annually. This number is rising and comprises more than 10% of newborns. Although complications of preterm birth are the main cause of death among children under 5 years of age, the survival rate has increased in developed countries [1]. Especially, the mortality rates of very-low-birthweight infants (VLBWs) have decreased, and strategies for follow-up have become essential [2].

After birth, VLBWs show a different growth pattern than that of term infants. Extrauterine growth restriction is common in VLBWs, and it may result from an interaction of various reasons: genetic and inadequate nutrition, morbidities affecting nutrient requirements, endocrine problems, and treatments [3,4]. Moreover, it is known that the composition of their body is different from that of term infants [5]. However, significant catch-up growth is usually shown during the first year of life, and differences in body composition reduce [5,6]. This pattern of postnatal growth is assumed to be associated with neurodevelopmental outcomes. Although advancements in brain imaging can predict neurodevelopmental outcomes, predicting developmental outcomes with growth measurement [7,8] would be beneficial for primary healthcare or in developing countries with low medical resources. Furthermore, it can be the first step for the research on promoting the growth of babies for their development. Despite efforts, there have been debates on which period (antenatal, during intensive care, or postdischarge) and which parts of the body (head circumference (HC), length, or weight) should be monitored as indicators of neurodevelopment [9,10,11,12].

Hence, this study aimed to find indicators for neurodevelopment of very preterm infants from their physical growth by evaluating the association between developmental outcomes and growth in the hospital and early period after discharge to home. In particular, we tried to find differences between appropriate-for-gestational-age infants (AGAs) and small-for-gestational-age infants (SGAs).

## 2. Materials and Methods

### 2.1. Patients and Data Collection

We conducted a retrospective cohort study of infants who were born at Seoul National University Children’s Hospital with birthweight under 1500 g or a gestational age prior to 32 weeks between 2009 and 2014. Among them, those who had Bayley Scales of Infant Development and Toddler Development, third edition (Bayley-III) around the corrected age (CA) of 18 months were included. Infants whose growth measurement data were not applicable or those who were diagnosed with congenital anomaly or cerebral palsy, which could result in severe neurodevelopmental problems, were excluded.

Data were collected from the infants’ hospital records. Gestational age was determined by first-trimester ultrasound and last menstrual period [13]. Trained nurses measured the infants in the neonatal intensive care unit and at the follow-up clinic. After birth, weight was measured daily, and crown–heel length and HC once a week until discharge. The baby was lying supine during height measurement, and HC was measured by a soft tapeline. The growth z-scores were estimated at the time of birth, at postmenstrual age (PMA) 35 weeks, at 4 months, and at 18 months CA by using the Fenton Preterm Growth Chart and World Health Organization measurements [14]. SGA was defined as <−2.0 SD at the time of birth, and extrauterine growth retardation was defined as the reduction in weight z-score between birth and at PMA 35 weeks >1.0 SD [15]. The differences in the z-scores for four time periods were calculated to estimate growth velocities: from birth to PMA 35 weeks, PMA 35 weeks to 4 months CA, 4 months to 18 months CA, and birth to 18 months CA. The Bayley-III was administered by a certified nurse at the follow-up clinic around CA of 18 months.

### 2.2. Statistical Analysis

To assess the relationship between 18-month Bayley-III scores and growth z-scores of each growth parameter at four time points, multivariate linear regression was performed. The association between the changes in z-scores of growth and Bayley-III scores was studied by using multivariate linear regression, adjusting for gestational age, birthweight, sex, respiratory distress syndrome (RDS), patent ductus arteriosus (PDA) treatment, bronchopulmonary dysplasia grades (none, mild, moderate, severe), intraventricular hemorrhage (IVH), periventricular leukomalacia (PVL), necrotizing enterocolitis (stage ≥ 2), sepsis, retinopathy of prematurity (ROP) operation, and maternal chorioamnionitis. Both analyses were performed for AGA and SGA infants. Analyses were conducted by using STATA version 15.1 (StataCorp, College Station, TX, USA).

## 3. Results

**Figure 1:**
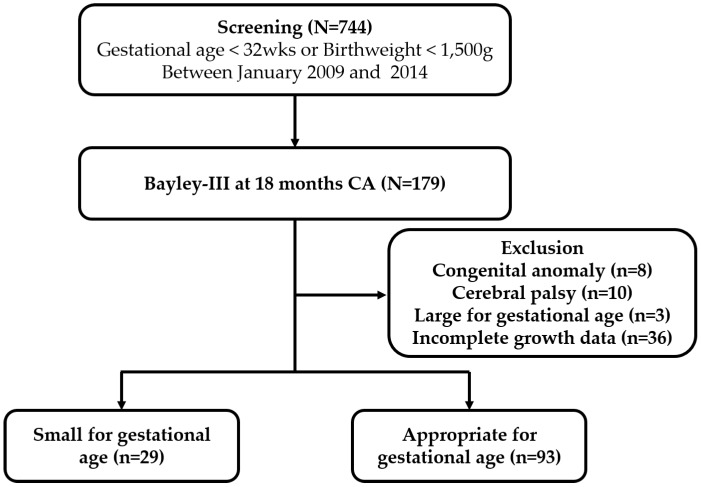
Flow diagram of the study population.

The study population comprised 122 infants born from 2009 to 2014 (Figure 1). The descriptive data, including perinatal factors and Bayley-III scores, are presented in Table 1. Among them, 29 (23.8%) were SGAs, and 93 (76.2%) were AGAs. AGAs were born two weeks earlier and underwent more ROP operations than SGAs. The z-scores of all the SGAs’ bodyweights were less than −2.0 SD at PMA 35 weeks. Otherwise, there were no significant differences in perinatal clinical factors between the two groups. The mean scores of Bayley-III in cognition were slightly higher in AGAs but not statistically significant, although language score was significantly higher in AGAs. The ratios of infants with Bayley-III scores of less than 85 were not different between the two groups.

**Figure 2:**
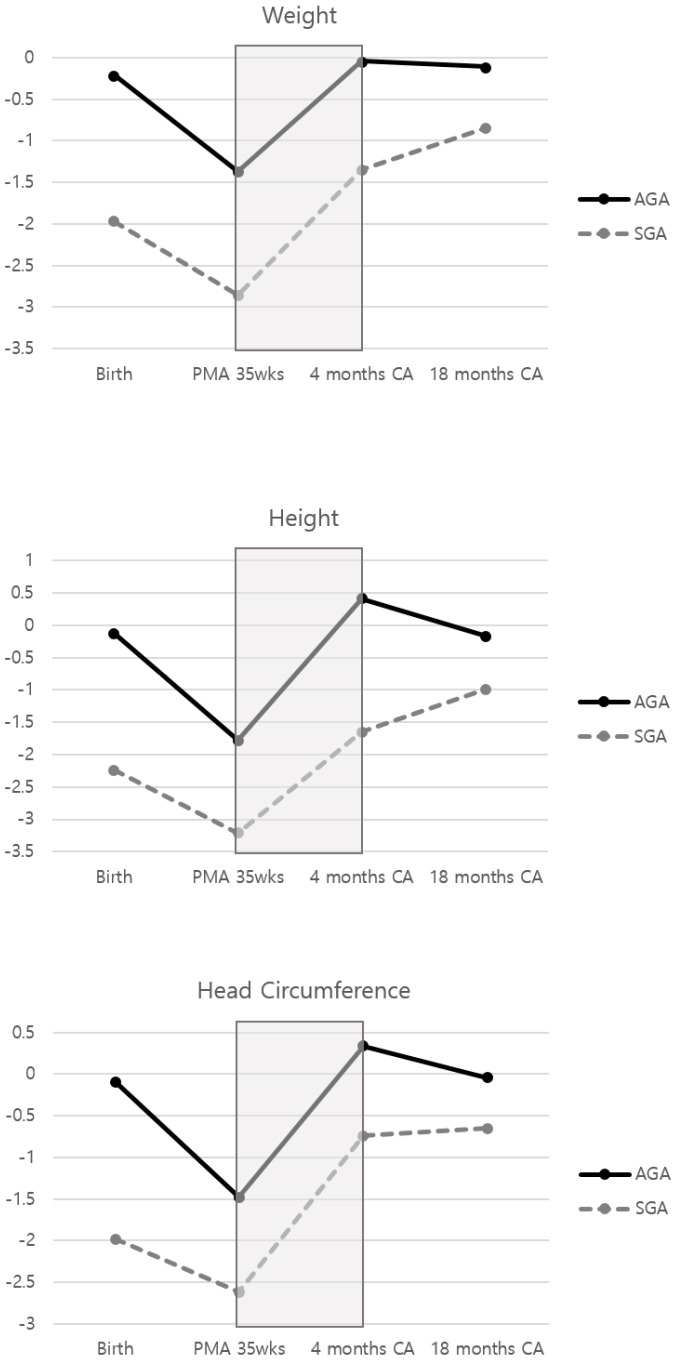
Infant size mean z-scores from birth to 18 months corrected age (CA). The mean z-score changes between postmenstrual ages 35 weeks and 18 months were not significantly different between small-for-gestational-age and appropriate-for-gestational-age infants (gray box).

Figure 2 shows growth patterns of both groups. The mean z-scores of all three growth indicators decreased from birth to PMA 35 weeks in both groups, and AGAs showed a significantly wider range of decrease (*p* < 0.05). From PMA 35 weeks to 4 months CA, the scores rose by similar amounts in both groups and exceeded the mean z-scores at birth in SGAs. From 4 months to 18 months CA, the patterns were different. The three indicators for SGAs continued to increase to 18 months CA; however, those for AGAs were similar or slightly lower (*p* < 0.05).

The z-score of birthweight showed a tendency toward correlation (*p* < 0.15) in most Bayley-III categories in both AGAs and SGAs, with the exception of motor development in SGAs. Additionally, the z-scores of HC at 4 months and 18 months CA mostly showed a positive correlation with three categories of Bayley-III in AGAs. The motor scale had tendencies to three growth indicators of AGAs in 18 months CA (Table 2).

The z-score changes in HC from birth to 18 months CA were associated with higher Bayley-III scores in cognition and motor development. Interestingly, when analyzed with subdivisions, only HC growth between PMA 35 weeks and 4 months CA had a positive correlation with Bayley-III scores in cognition and motor development. Growth before PMA 35 weeks or after 4 months CA was not associated with scores. The z-score changes in weight and length from birth to 18 months CA were also associated with a higher Bayley-III score in the motor scale (Table 3).

For SGAs, the z-score of birthweight was the only factor that was associated with improved neurodevelopmental outcomes. The z-scores of weights at other follow-up visits, other growth indicators, or the z-score changes were not correlated with Bayley-III in SGAs (Table 2 and Table 3).

## 4. Discussion

The catch-up growth of HC was associated with better neurodevelopmental outcomes in the study population. The z-scores of three growth indicators dropped from birth to PMA 35 weeks, although these recovered to the level at the birth for 5 months (PMA 35 weeks to 4 months CA). Thus, head growth in this recovery phase is strongly associated with improved cognitive and motor outcomes especially in very preterm AGA infants. Growth from birth to 18 months CA was also correlated with improved motor outcomes in AGAs. In contrast, SGAs showed different growth patterns, and the phase was not associated with neurodevelopmental outcomes.

Although several studies suggested that head growth is associated with development [9,10,16,17,18,19], only a few studies emphasized the importance of growth in the early period after term equivalent age as our study did [10,16]. In our study, HC at 4 months CA in the AGA group exhibited a positive correlation with all three domains of cognitive, language, and motor development at 18 months CA. These relationships were reproduced with HC at the time of the Bayley-III test. Furthermore, the z-score change analysis elucidated that HC growth between PMA 35 weeks and 4 months CA had contributed to these developmental benefits in cognitive and motor development, while other periods did not. According to previous studies, experience-dependent synapse formation begins at the late fetal (1 month before birth) and early neonatal life. During the period, neurogenesis associated with seeing, hearing, language, high cognitive function, and memory rapidly increases. The motor cortex also begins its rapid development after PMA 40 weeks [20,21]. The biological importance of this period is highly correlated with our results. Furthermore, there are several reasons why head growth should be monitored rather than HC at birth. First, even though skilled professionals perform the measurements, it is difficult to accurately measure the HC at birth due to cephalohematoma or instruments. Second, postnatal growth reflects the effects of the postnatal environment, such as neonatal disease, while HC at birth reflects the effects of maternal environmental conditions, such as placental insufficiency [10].

In this study, increased weight between birth and PMA 35 weeks was also associated with language development, and growth between birth and 18 months CA was related to motor development. Several studies reported weight and linear growth as growth indicators for neurodevelopmental outcomes. Other studies found that cognitive development was associated with birthweight and increase of weight from birth to discharge [16,22]. Leppänen et al. also showed that weight growth between birth and 2 years CA correlated to full-scale IQ at 5 years of age in non-SGA children [11]. These studies emphasized the importance of designing nutritional support after discharge but also noted that adverse effects of overnutrition, such as later overweight and higher blood pressure, should be considered [22]. Linear growth of preterm infants after birth is another indicator that is reported to be associated with developmental outcomes [21,23]. The authors speculated that preterm infants with linear growth suppression may have inappropriately low IGF-1 levels, which are important for linear growth, neuronal growth, and differentiation [21].

Moreover, there were different results between the SGA and AGA groups. Good HC growth around term age was especially associated with good developmental outcomes in the AGA group, while there was no significant result in the SGA group. This can result from discordant growth patterns between the two groups. In this study, the mean z-score of HC in SGAs increased from PMA 35 weeks to 18 months CA, whereas those of AGAs were constant from 4 months to 18 months CA. Birthweight was the only indicator that correlated with development in SGAs, and the degree of insults before birth may be among the most important factors in this group. While growth from birth to 18 months CA and development of cognition showed a tendency toward correlation (*p* < 0.15) and some previous studies reported that good HC growth from birth to CA of 4 months was associated with improved cognition in SGA infants, further research with a larger number of SGA infants is needed [11].

The strengths of this study include results from 122 very preterm infants’ neurodevelopment and detailed analysis of the implications of SGA and head growth during distinct time periods. Second, a single certified nurse consistently performed the Bayley-III test with an identical protocol in a single center. However, it is important to note some limitations. First, this study could not analyze which factors of exposure after discharge (such as breastfeeding period, interventions to improve the neurodevelopment, and nutritional support) may affect the growth and development of preterm infants because of limited data. Second, to obtain enough numbers of the study population, we used both gestational age (<32 weeks) and birthweight (<1500 g) for defining the cohort. There were 15 patients (12.3%) who were born after gestational age 32 weeks and after, who might experience a lesser number of neonatal morbidities. However, their neurodevelopmental outcomes were not different compared to babies who were born earlier (data not shown). In addition, we adjusted outcomes with gestational age and birthweight to reduce confounding. Lastly, we could not adjust neurodevelopmental outcomes with socioeconomic status of families. Further, there are some previous studies that have been published with the same limitation [19,21].

## 5. Conclusions

We have shown that the HC at 4 months CA was an important indicator of favorable neurodevelopmental outcomes in preterm infants, and head growth spurt occurring between PMA 35 weeks and 4 months CA contributed to these benefits. Since the growth pattern was different depending on whether they were SGAs or AGAs, the period and indices to be monitored should be set differently for AGAs and SGAs. Further studies evaluating factors affecting head growth are needed to develop appropriate strategies for catch-up growth.

## Figures and Tables

**Table 1:** Characteristics and perinatal morbidities of the study population.

	SGA ^1^ (*n* = 29)	AGA ^2^ (*n* = 93)	*p* Value
	Mean (SD) or *n* (%)	Mean (SD) or *n* (%)	
Gestational age, weeks day/7	30 4/7 (2 6/7)	28 1/7 (2 2/7)	<0.001
<26	3 (10.3)	14 (15.1)	<0.001
26–28	5 (17.2)	45 (48.4)	
29–31	9 (31.0)	31 (33.3)	
≥32	12 (41.4)	3 (3.2)	
Primiparous	20 (69.0)	69 (74.2)	0.580
Caesarean section	24 (82.8)	51 (54.8)	0.007
Multiple birth	12 (41.4)	51 (54.8)	0.205
Female	15 (51.7)	46 (49.5)	0.832
Birthweight, g	892.7 (271.6)	1053.2 (296.5)	0.010
Length, cm	34.1 (3.5)	36.0 (3.5)	0.014
Head circumference, cm	24.9 (2.4)	25.4 (2.5)	0.404
Maternal chorioamnionitis	3 (10.3)	14 (15.1)	0.523
Respiratory distress syndrome	10 (34.5)	49 (52.7)	0.087
Patent ductus arteriosus treatment			
Medication	10 (34.5)	46 (49.5)	0.050
Operation	1 (3.5)	12 (12.9)	
Bronchopulmonary dysplasia			
Mild	7 (24.1)	30 (32.6)	0.100
Moderate	6 (20.7)	29 (31.5)	
Severe	1 (3.5)	8 (8.7)	
Intraventricular hemorrhage ≥ Grade III	1 (3.5)	4 (4.3)	0.840
Periventricular leukomalacia	1 (3.5)	3 (3.2)	0.953
Necrotizing enterocolitis ≥ Stage 2	4 (13.8)	10 (10.8)	0.654
Sepsis	2 (9.5)	16 (20.3)	0.255
Retinopathy of prematurity operation	4 (19.1)	34 (43.0)	0.044
Extrauterine growth retardation	12 (41.4)	54 (58.1)	0.115
Cognition score			
Mean (SD)	97.4 (10.8)	99.3 (13.3)	0.488
<85	2 (6.9)	7 (8.5)	0.910
Language score			
Mean (SD)	90.6 (11.9)	97.0 (15.5)	0.043
<85	9 (31.0)	21 (22.6)	0.356
Motor score			
Mean (SD)	97.1 (9.1)	96.8 (11.8)	0.905
<85	2 (6.9)	12 (12.9)	0.376

^1^ Small-for-gestational-age. ^2^ Appropriate-for-gestational-age.

**Table 2:** Correlations between growth indicators after birth and 18-month Bayley Scores.

Growth Indicators	Time	Cognition			Language					Motor			
		SGA (*n* = 29) ^1^	AGA (*n* = 93) ^2^		SGA			AGA		SGA		AGA	
		ß	*p*	ß	*p*	ß	*p*	ß	*p*	ß	*p*	ß	*p*
Weight	Birth	12.78	0.048	3.61	0.091	13.19	0.059	4.79	0.057	6.16	0.232	4.15	0.018
	35 weeks	11.61	0.214	3.99	0.157	12.75	0.234	9.19	0.004	6.32	0.407	2.15	0.348
	4 months CA ^3^	2.04	0.402	1.01	0.453	4.00	0.141	1.53	0.329	0.56	0.777	2.08	0.053
	18 months CA	2.92	0.209	0.30	0.841	3.15	0.236	−0.48	0.780	1.78	0.347	2.70	0.022
Length	Birth	2.91	0.522	−1.94	0.356	−1.60	0.759	−0.64	0.793	1.19	0.747	−1.62	0.339
	35 weeks	1.99	0.772	−2.63	0.292	−0.47	0.953	1.93	0.507	−0.88	0.874	0.51	0.803
	4 months CA	0.90	0.706	−0.23	0.821	3.52	0.183	−0.85	0.478	0.00	0.999	0.42	0.609
	18 months CA	2.08	0.304	−0.23	0.867	1.53	0.511	−0.60	0.706	0.76	0.646	1.69	0.124
Head Circumference	Birth	−4.27	0.458	0.36	0.822	−4.41	0.504	3.75	0.040	−5.27	0.249	−0.62	0.628
	35 weeks	4.29	0.586	1.81	0.328	0.71	0.938	3.49	0.104	2.59	0.683	1.70	0.255
	4 months CA	0.91	0.739	2.71	0.012	1.20	0.702	2.26	0.076	−0.77	0.727	2.38	0.006
	18 months CA	4.57	0.207	3.55	0.003	4.42	0.290	3.08	0.030	1.41	0.637	2.60	0.008

Adjusted for gestational age, birthweight, sex, respiratory distress syndrome, patent ductus arteriosus treatment, bronchopulmonary dysplasia grades, intraventricular hemorrhage grade III, periventricular leukomalacia, necrotizing enterocolitis (stage ≥ 2), sepsis, retinopathy of prematurity operation, and maternal chorioamnionitis. ^1^ Small–for-gestational-age. ^2^ Appropriate-for-gestational-age. ^3^ Corrected age

**Table 3:** Correlations between growth from birth to 18 months of corrected age and 18-month Bayley Scores.

Growth Indicators	Time Period	Cognition				Language				Motor			
		SGA (*n* = 29) ^1^		AGA (*n* = 93) ^2^		SGA		AGA		SGA		AGA	
		ß	*p*	ß	*p*	ß	*p*	ß	*p*	ß	*p*	ß	*p*
Weight	Birth to 18 months CA ^3^	2.53	0.309	−0.23	0.878	2.06	0.474	−0.69	0.685	1.62	0.421	2.40	0.040
	Birth to 35 weeks	0.68	0.942	2.68	0.407	−8.12	0.438	10.90	0.003	0.81	0.914	1.68	0.520
	35 weeks to 4 months CA	1.29	0.602	0.11	0.934	3.20	0.249	−0.57	0.723	0.14	0.942	1.66	0.132
	4 months CA to 18 months CA	0.99	0.674	−1.42	0.438	−0.59	0.829	−3.58	0.092	1.23	0.514	0.27	0.855
Length	Birth to 18 months CA	1.43	0.475	0.51	0.688	1.78	0.435	−0.28	0.849	0.50	0.759	2.04	0.044
	Birth to 35 weeks	−2.56	0.617	0.06	0.973	1.76	0.764	1.51	0.477	−1.99	0.629	1.49	0.312
	35 weeks to 4 months CA	0.65	0.784	0.21	0.838	3.50	0.181	−1.16	0.329	0.11	0955	0.33	0.685
	4 months CA to 18 months CA	3.14	0.298	0.16	0.901	−2.42	0.486	0.78	0.599	1.69	0.492	0.81	0.431
Head Circumference	Birth to 18 months CA	5.35	0.102	2.20	0.027	5.27	0.164	0.53	0.653	3.02	0.263	1.97	0.040
	Birth to 35 weeks	6.56	0.247	0.72	0.598	4.77	0.468	−0.89	0.579	6.63	0.140	1.39	0.207
	35 weeks to 4 months CA	0.38	0.887	2.62	0.032	1.08	0.727	1.30	0.367	−1.04	0.629	2.26	0.022
	4 months CA to 18 months CA	1.61	0.561	0.27	0.885	1.23	0.698	0.50	0.817	1.57	0.479	−0.92	0.540

Adjusted for gestational age, birthweight, sex, respiratory distress syndrome, patent ductus arteriosus treatment, bronchopulmonary dysplasia grades, intraventricular hemorrhage grade III, periventricular leukomalacia, necrotizing enterocolitis (stage ≥ 2), sepsis, retinopathy of prematurity operation, and maternal chorioamnionitis. ^1^ Small-for-gestational-age. ^2^ Appropriate-for-gestational-age. ^3^ Corrected age.
